# Quantifying the Epidemiological Impact of Vector Control on Dengue

**DOI:** 10.1371/journal.pntd.0004588

**Published:** 2016-05-26

**Authors:** Robert C. Reiner, Nicole Achee, Roberto Barrera, Thomas R. Burkot, Dave D. Chadee, Gregor J. Devine, Timothy Endy, Duane Gubler, Joachim Hombach, Immo Kleinschmidt, Audrey Lenhart, Steven W. Lindsay, Ira Longini, Mathias Mondy, Amy C. Morrison, T. Alex Perkins, Gonzalo Vazquez-Prokopec, Paul Reiter, Scott A. Ritchie, David L. Smith, Daniel Strickman, Thomas W. Scott

**Affiliations:** 1 Department of Epidemiology and Biostatistics, Indiana University Bloomington School of Public Health, Bloomington, Indiana, United States of America; 2 Fogarty International Center, National Institutes of Health, Bethesda, Maryland, United States of America; 3 Department of Biological Sciences, Eck Institute for Global Health, University of Notre Dame, Notre Dame, Indiana, United States of America; 4 Centers for Disease Control and Prevention (CDC), San Juan, Puerto Rico; 5 Australian Institute of Tropical Health and Medicine, James Cook University, Cairns, Queensland, Australia; 6 Department of Life Sciences, Faculty of Science and Agriculture, The University of the West Indies, St. Augustine Campus, St. Augustine, Trinidad and Tobago; 7 QIMR Berghofer Medical Research Institute, Brisbane, Queensland, Australia; 8 Department of Medicine, Upstate Medical University of New York, Syracuse, New York, United States of America; 9 Signature Research Program in Emerging Infectious Disease, Duke-NUS Medical School, Singapore; 10 Initiative for Vaccine Research, World Health Organization, Geneva, Switzerland; 11 Tropical Epidemiology Group, Department of Infectious Disease Epidemiology, London School of Hygiene and Tropical Medicine, London, United Kingdom; 12 Department of Pathology, School of Health Sciences, University of Witwatersrand, Johannesburg, South Africa; 13 Centers for Disease Control and Prevention, Center for Global Health/Division of Parasitic Diseases and Malaria/Entomology Branch, Atlanta, Georgia, United States of America; 14 School of Biological and Biomedical Sciences, Durham University, Durham, United Kingdom; 15 Department of Biostatistics, University of Florida, Gainesville, Florida, United States of America; 16 Innovative Vector Control Consortium; 17 Department of Entomology and Nematology, University of California, Davis, California, United States of America; 18 Department of Environmental Studies, Emory University, Atlanta, Georgia, United States of America; 19 Department of Medical Entomology, Institut Pasteur, Paris, France; 20 College of Public Health, Medical, and Veterinary Sciences, Australian Institute of Tropical Health and Medicine, James Cook University, Cairns, Queensland, Australia; 21 Institute for Health Metrics and Evaluation, University of Washington, Seattle, Washington, United States of America; 22 Bill & Melinda Gates Foundation, Seattle, Washington, United States of America; University of California, Berkeley, UNITED STATES

## Introduction

Dengue virus (DENV) is a self-limiting illness in tropical and subtropical regions around the globe caused by four closely related, but distinct, virus serotypes (DENV-1, -2, -3, and -4) that are transmitted among humans by mosquitoes, primarily *Aedes aegypti* [1]. Approximately 4 billion people living in more than 128 countries are at risk of infection [2]. Each year there are an estimated 400 million new infections, of which about 100 million manifest as apparent illness [3]. The outcome of human infections ranges from asymptomatic to mild illness to severe, life-threatening disease [4]. DENV not only causes more human morbidity and mortality than any other arthropod-borne virus but it is also a growing public health threat. There has been a dramatic 4-fold increase in dengue cases between 1990–2013 and dengue continues to expand in geographic range [2,3,5,6].

Presently, vector control is the primary means for preventing dengue [7]. Several vaccine constructs are in clinical trials and initial results are encouraging [8]; recently licensure was granted for the Sanofi Pasteur vaccine in Mexico, Brazil, and the Philippines [9]. A few well-documented successes indicate that, when rigorously applied, vector control can reduce dengue. The advent of DDT in 1947 led to a hemisphere-wide program in the 1950s and 1960s across Central and South America that dramatically reduced *Ae*. *aegypti* populations, resulting in impressive reductions in yellow fever and dengue [10]. During the 1970s–1980s [11] and the 1980s–1990s [12], respectively, Singapore and Cuba successfully used vector control and larval source reduction to reduce the force of DENV infection (i.e., per capita risk of human infection [13]) and, thus, disease. Recent trials of indoor residual spraying [14] and indoor space spraying [15] appeared to reduce human DENV infections. Regrettably, these control achievements were rare and ultimately transient. Dengue reinvaded Latin America after the *Ae*. *aegypti* eradication campaign ended, rebounded in Singapore and Cuba after 20 and 16 years of successful control, respectively, and is increasingly being reported in Africa due to improved surveillance [16].

Although the concept of dengue vector control seems straightforward, successful broad-scale application has been difficult to achieve and even harder to sustain [17]. In most settings, dengue vector control failed to prevent epidemics and it is not slowing expansion of the virus’s geographic range [3,17,18]. Unsuccessful control programs are often attributed to inadequate responses to a robust virus transmission system. Outbreaks may occur due to combinations of risk factors, including expanding *Ae*. *aegypti* populations, virus and mosquito dispersal via extensive human travel networks, weak vector control infrastructure, lack of resources to mount effective interventions, lack of political will, and ineffective implementation of existing tools and strategies [17,19]. A recent review concluded that dengue vector control can be effective, but only when implementation is expedient, comprehensive, and sustained [7].

Despite these major challenges, there is growing interest in combining vector control with vaccination once a dengue vaccine becomes widely available, which recognizes that one intervention is insufficient to effectively reduce the burden of disease. Theoretically, a dengue vaccine could elevate herd immunity, making it easier to sustain the effects of vector control on virus transmission. Similarly, vector control could lower the force of DENV infection, making it easier to achieve vaccine delivery goals [20]. Results from studies with malaria [21,22] and lymphatic filariasis [23] support the impact of simultaneously targeting vectors and pathogens.

The next critical step is selecting vector control strategies that are best suited for combining with a vaccine. Selection criteria will likely depend on local dengue ecologies. Some funding agencies are responding by enabling investigators and developers to carry out quantitative and epidemiologic assessments of novel approaches, e.g., release of *Wolbachia*-infected *Ae*. *aegypti* [24,25], spatial repellents and vapor active insecticides [26,27], and enhanced community mobilization [28] as part of early-phase intervention evaluations [24]. Surprisingly, most existing dengue vector control strategies (e.g., larvicides and outdoor versus indoor insecticide space spraying) have not been robustly evaluated for impact on reducing human infection and disease [29,30]. Some trials have evaluated entomological impact [31], but reductions in mosquito populations do not correlate well with predictable reductions in dengue disease [20,32]. Along with underpowered and inefficient control responses, the fact that current dengue vector control tools and strategies lack quantitative evidence of efficacy from field trials helps explain why contemporary control programs fail more often than they succeed.

A Partnership for Dengue Control-sponsored workshop was convened to begin to address this gap [33]. A panel of international experts identified the vector control tools currently available that may have the highest probability of success in reducing dengue and field trial experimental design attributes necessary to assess their efficacy. Although vaccines will likely be implemented concurrently with vector control, before that can be done epidemiological trials are needed to quantify the protective efficacy of vector control interventions alone. Results will provide a benchmark for subsequent trials in which combinations of interventions are assessed. This approach builds on recent calls for increased rigor in the design of vector control studies [34] with an emphasis on dengue. In addition to benefiting dengue control programs, the results of the proposed trial described herein will be relevant for prevention of other *Ae*. *aegypti*-borne viral infections of increasing public health importance, such as chikungunya [35] and Zika [36] viruses.

## Dengue Vector Control Experimental Design Considerations

### Ecological Complexities

*Aedes aegypti* will be the primary target of our intervention. Other mosquito species such as *Aedes albopictus* and *Aedes polynesiensis* can play secondary roles in DENV transmission in specific geographic areas [37], but a trial site where *Ae*. *aegypti* is the only vector species present is desirable to simplify interpretation of trial results. Given the overlapping distribution of *Ae*. *aegypti* and other vectors of DENV, especially *Ae*. *albopictus*, if this is not achievable, the difference in ecology across the species present must be taken into account when selecting control methods, intervention application locations, and in the interpretation of results. If a site where only *Ae*. *aegypti* is present is identified, trials may need to be repeated where other vector species are present.

DENV transmission is local, focal, heterogeneous, and highly efficient [38,39]. Transmission is facilitated by *Ae*. *aegypti*, which live in relatively low densities in close association with humans and seldom fly farther than 100 m [40]. Transmission foci are connected at short distances by a combination of human and mosquito movement patterns and at longer distances by human movement alone [38,41]. Clusters of mosquitoes and the houses they infest (high-density hotspots) are highly focal, seldom larger than 30 m, and, even though they can consistently be detected, they are temporally unstable, i.e., fine-scale mosquito abundance is continually shifting across time and space [42]. Because *Ae*. *aegypti* is a day-biting mosquito, people are at risk of infection both in their homes and when they leave home. We therefore recommend (1) accounting for human movement in a cluster-randomized controlled trial (RCT) study design by either applying means to measure movement patterns during the follow-up period or focusing on a least mobile section of the population and (2) conducting intervention trials in areas with historically high levels of human infection rather than attempting to identify and attack mosquito hotspots.

### Possible Control Tools

Given *Ae*. *aegypti*’s peri-domestic habits, key candidate tools for vector control are those based on the use of long-lasting formulations of synthetic pyrethroids applied to walls, curtains, window screens, and water container covers [7]. Reduction of larval sources through either container removal or applications of insecticides or biological agents can decrease adult mosquito production [31], but mosquitoes often exploit cryptic containers that remain untreated without extensive insecticide fogging. To be most effective, larval control needs to be combined with methods targeting adult mosquitoes [7]. Recent evidence suggests that insecticide-treated curtains, window screens, and covers on water-holding containers can reduce *Ae*. *aegypti* densities in and around treated areas [43–45], indicating that these tools should be considered in an integrated approach. Innovative vector control tools that are currently being evaluated for malaria should be explored for dengue.

### Defining Impact

**Fig 1:**
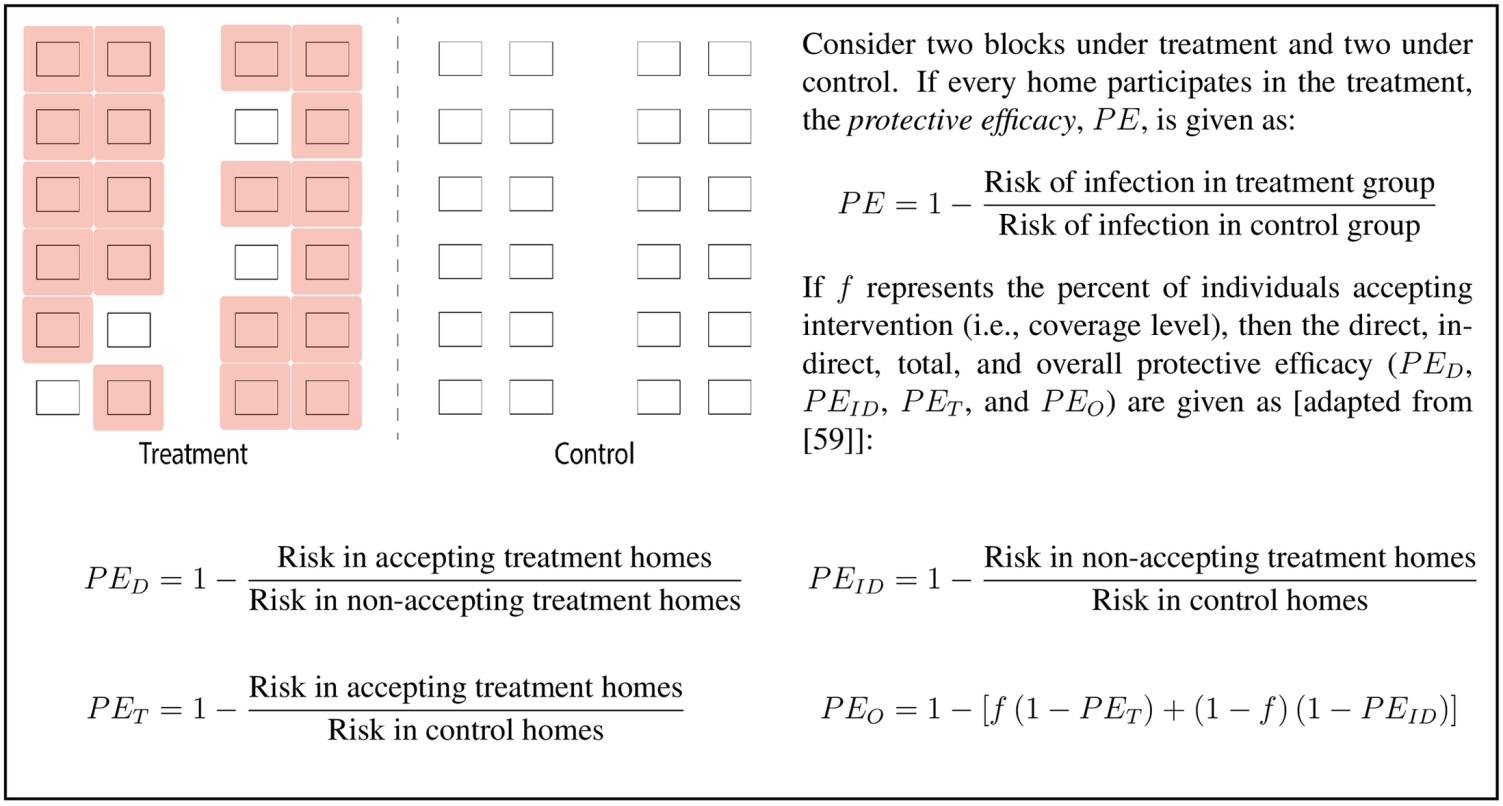
Protective efficacy: basic definitions.

The protective efficacy (PE) of a treatment relates the risk of infection within a group that receives the treatment to that of an untreated control group (see Fig 1). During the initiation of the trial, special messaging will be used to maximize the participation rate. By accounting accurately for individuals (or homes) within treatment areas that decline to participate in the trial, but still provide data on infection status, the relative direct and indirect effects of the trial can be calculated (Fig 1). Classical models originally developed for malaria [46] and subsequently applied to other mosquito-borne pathogens [47] offer a range of metrics for intervention trials (e.g., vectorial capacity, entomological inoculation rate, and basic reproductive number [48]) and a means for mathematically connecting those metrics with other measurable outcomes (e.g., attack rates and the force of infection [49]). These relatively simple mathematical associations offer an effective way to explore the expected relative impacts of an intervention with a given metric, e.g., a 50% reduction in larval habitats can lead to a 75% reduction in vectorial capacity [50]. After empirically-driven estimates of the impact of control strategies have been calculated from an RCT, the mathematical form of these dynamics will help identify metrics most likely to track transmission intensity and support the recommendation of appropriate combinations of interventions to achieve maximum reduction in infection and disease.

### Measuring Outcomes

Vector control is designed to directly impact mosquito populations. Although the primary endpoints of the proposed study are epidemiological, there are several crucial reasons why mosquito populations should be measured. In addition to continual testing for insecticide resistance in adults and larvae, longitudinal monitoring of immature and adult mosquito densities are necessary to confirm that the intervention affected the mosquito population as expected, and adult mosquito data can be used to assess the relative transmission risks inherent in each cluster. If mosquito densities vary in space so that some locations systematically over- or under-produce compared to other locations, population dynamics should be characterized before the trial begins and used to stratify randomization of treatment and control clusters.

Epidemiologic impact will be determined using three complementary approaches: longitudinal cohorts, febrile surveillance, and geographic clusters. Seroconversion by participants in prospective longitudinal cohorts is one way to accurately detect differences in human DENV infection and relative risk of infection [24,51]. Identifying seroconversions to tertiary or quaternary infections is difficult, thus a pediatric cohort in endemic settings would be advantageous to maximize transmission detection (i.e., people who enter the study as immunologically naïve or have a monotypic antibody response) and minimize the potential for movement between treatment and control study arms. Serostatus will be monitored to determine if there was more than one infection between any two tests. At locations where more than one flavivirus is transmitted (e.g., yellow fever, Japanese encephalitis, Zika) or where people have been vaccinated against other flaviviruses (e.g., yellow fever, Japanese encephalitis), interpretation of DENV seroconversions will need to account for cross-immunity to closely related viruses. If a trial is conducted in concert with, or soon after, administration of a DENV vaccine, the design can include a surrogate endpoint of clinically apparent DENV infections that are virologically confirmed, similar to what has been done in recent dengue vaccine trials [52]. A serologic-based endpoint could be used for vaccinated populations if, in the future, new assays are developed that can differentiate the immune response to vaccination versus a natural DENV exposure.

Within a subset of identified neighborhood clusters, participants will be recruited for a longitudinal cohort. Routine febrile surveillance consisting of one to three visits per household per week of people living near cohort participants will allow longitudinal comparisons of people with documented dengue illness [38]. Geographic cluster studies that screen people living within a designated radius (~100m) of a person with a laboratory-diagnosed DENV infection (the index case) will measure variation in fine-scale spatial patterns of DENV transmission [24]. Depending on how movement is accounted for or incorporated into the trial design, cohort sample size estimates will be in the range of 2,000 to 3,000 participants [51], with at least five times that number under febrile surveillance [38]. The number of clusters in the study will depend on background transmission, local herd immunity, anticipated effect size of the intervention, between cluster variation, and logistics capacity.

### Design and Analysis

**Fig 2:**
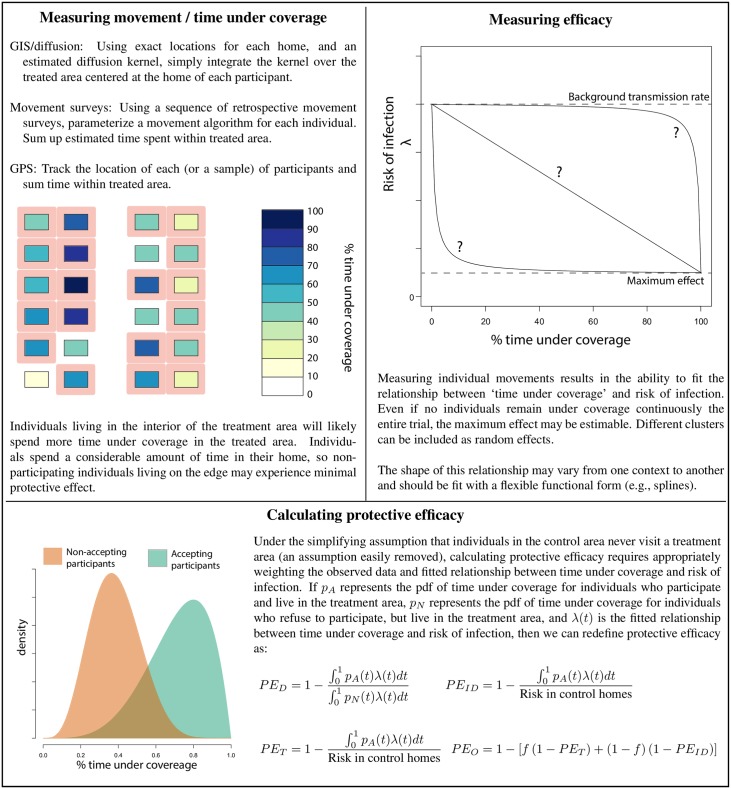
Estimating protective efficacy when considering movement out of coverage areas.

Given the diffusive effect of mosquito dispersal on transmission risk, vector control trials typically employ a cluster RCT study design [53] in which communities are randomly allocated to intervention or control arms [34]. One of the largest challenges with measuring the impact of vector control on dengue transmission, as opposed to other mosquito-borne diseases such as malaria, is that dengue vectors bite during the day. People who live within a cluster assigned to receive the intervention may, therefore, spend a considerable amount of their day at risk of infection in untreated areas. Conversely, those living in untreated areas may move into treated areas during their daytime activities. To estimate accurately the effectiveness of vector control on dengue transmission, information on the movement patterns of individuals within the treatment and control arms is needed to estimate individual-level time under coverage. One way to limit the effect of human movement is to have clusters that include large geographic areas and to enroll children who may not be as mobile as adults, although this may be operationally difficult to achieve and expensive. The effectiveness of a community-applied strategy, as often occurs in vector control, will likely only be fully felt by those who never leave the treated area (see Fig 2). As an individual spends increasingly more time in locations where transmission continues unhindered, their individual risk of infection recovers to background levels. By incorporating individual-level time under coverage in analyses, the maximum possible effect (as well as the average predicted effects) of the intervention can be estimated (see Fig 2). When possible, if movement patterns systematically differ across cluster arms, random assignment of clusters to treatment versus control should be stratified by movement level, much like cluster stratification using baseline incidence or prevalence rates commonly employed in malaria trials [54].

An alternate design to the “dose-response” approach described above would focus on only those individuals who move the least. Initial movement surveys may identify a sub-population that is less mobile, i.e., young children. Clusters can then be defined based on movement range and design, and analysis can use the standard metrics described in Fig 1. This approach might necessitate large numbers of participants in clusters if fewer susceptible individuals qualify for enrollment. On the other hand, this approach directly assesses treatment with a more rigorous, but potentially less valid, assumption of equal exposure to all those who are treated. The choice as to which study design is optimal will likely be context and resource dependent. If the more rigorous option is taken, movement data and analyses metrics in Fig 2 can be applied as a secondary endpoint.

### Site Selection

Trial site selection will follow a detailed process of criteria evaluation to identify locations with the capacity to rigorously assess the intervention [34]. DENV must be endemic with existing information on transmission intensity and local *Ae*. *aegypti* population dynamics. Infrastructure and trained personnel must be in place for measuring entomological and epidemiological outcomes. Community engagement of study participants should be ongoing throughout the entire trial period. Public opinion and compliance will be key for a successful assessment. A framework for communicating with stakeholders has been developed for evaluating genetically modified mosquitoes, a strategy in which public opinion and compliance are challenging and from which trials using other vector control interventions could benefit [55].

Preliminary studies on opinions about vector control will help inform the most effective strategy. Local *Ae*. *aegypti* populations will be screened for resistance to determine available insecticides for each potential site. Baseline virology and vector sampling should be carried out for one year before interventions and intervention should last for at least two transmission seasons to account for inter-annual variation in transmission and mosquito population densities. It will be important for funders to understand the return on investment of longer duration trials for informed decision-making.

## Concerns

### Insecticide Resistance

Insecticide resistance is a particularly worrisome challenge for dengue prevention because most dengue vector control strategies rely heavily on the use of chemical control. The resistance status of the target vector population should be taken into consideration when designing interventions to ensure that the product used will not only achieve maximum impact but have sustained effects throughout anticipated DENV transmission cycles. Resistance should be monitored throughout the trial, e.g., by knockdown resistance (kdr) and WHO bioassays [56]. Given the extended duration of continual insecticide application, options to mitigate selection pressure should be determined prior to intervention roll-out to avoid, mitigate, and minimize the development of resistance associated with the specific interventions. The degree to which insecticide resistance compromises dengue vector control efficacy remains largely unknown, but should not be underestimated given that the most cost-effective vector control tools rely heavily on the use of a highly limited number of public health insecticides [57].

### Ethics

The ethical, social, and cultural framework for trial site selection outlined by Lavery et al. [58] will be applied. As such, community authorization and participation is a priority for site selection and conducting a meaningful trial. After the regulatory process has been defined, thorough ethical evaluation of the study design will be carried out, including the possible use of a placebo if no established intervention is used. During the trial, government and public health dengue control programs must be allowed to conduct routine vector control activities because this cannot ethically be prevented or hindered. Trial management should follow fundamentals used for Good Clinical Practice, including standard operating procedures; a trial steering committee; convening or engaging data monitoring, safety, and ethics committees; a dedicated trial monitor; and that the analytical plan should be finalized before commencement of the trial.

## Recommendation

The “local and focal” nature of DENV transmission and the often large variation in transmission patterns from year to year, combined with the short-range dispersal of the day-biting vector *Ae*. *aegypti*, create a challenging context for evaluating the effectiveness of any dengue vector control intervention. Detecting a causal relationship between entomological impact and a reduction in dengue transmission intensity requires careful trial design. Within the design of an RCT, it is important to account for human movement patterns. If human movement is ignored, a successful intervention may appear to have failed. Ignoring human movement during study design considerations would require an artificial increase in the expected effectiveness to achieve adequate power because every individual in a treatment cluster will be predicted to have the “maximum” effect. Ignoring human movement during analysis would almost certainly reduce the ability to detect a significant effect of the intervention due to spill-over of “treated” individuals into areas without protection. Potential confounding factors such as the development of insecticide resistance over the course of the trial or an unanticipated lack of residual effect of the intervention could directly compromise trial outcomes and should be closely monitored. Building a bridge between dengue control experiences and those of other vector-borne pathogens, especially *Plasmodium*, may accelerate advancement of control techniques across multiple diseases.

## Conclusions

Very few vector control tools for any mosquito-borne pathogen have been assessed in Cochrane-style reviews, which are generally considered the most rigorous assessment of health interventions, largely due to design flaws [34]. For dengue, there are very few published studies [28] and even fewer studies that formally assess the impact of existing insecticide-based strategies on dengue. Although there is progress in the development of a dengue vaccine, it is likely for the foreseeable future that integrating vector control and vaccination will be necessary [33]. Integrated Vector Management is endorsed by the World Health Organization for the control of dengue and other vector-borne diseases [57]. Insecticides cannot be distributed and disseminated in vast quantities across broad geographic areas without concern for ecological and environmental impact. Subjecting any population to continued exposure to insecticides requires precise estimates of both the costs (economic and environmental) and the public health benefits.

As dengue’s global burden grows, the need for proven effective vector control options will increase. We argue that quantifying the epidemiological impact of any vector control intervention on DENV transmission will require assessments of human movement. We propose two options: cluster sizes can be enlarged and maximum age of participants can be reduced until protection is approximately uniformly felt by all those who are intended to be treated. This may, however, greatly increase required resources for already economically challenging trials. The alternative option is more efficient, but comes with some cost in rigor. As described in Fig 2, human movement patterns can be explicitly incorporated into the calculations for protective efficacy, with movement either inferred from simple geographic-based movement kernels or explicitly estimated by extrapolating the movement patterns of a sub-population within each cluster. Using either approach, intervention trials can provide robust and meaningful information. Insights from such trials will help guide the scaling up of effective dengue control strategies, whether vector control alone or in combination with vaccines, and will be applicable to other *Ae*. *aegypti*-borne viral infections of current public health concern, such as chikungunya and Zika viruses.
